# First-in-Humans Study of ^68^Ga-DOTA-Siglec-9, a PET Ligand Targeting Vascular Adhesion Protein 1

**DOI:** 10.2967/jnumed.120.250696

**Published:** 2021-04

**Authors:** Riikka Viitanen, Olli Moisio, Petteri Lankinen, Xiang-Guo Li, Mikko Koivumäki, Sami Suilamo, Tuula Tolvanen, Kirsi Taimen, Markku Mali, Ia Kohonen, Ilpo Koskivirta, Vesa Oikonen, Helena Virtanen, Kristiina Santalahti, Anu Autio, Antti Saraste, Laura Pirilä, Pirjo Nuutila, Juhani Knuuti, Sirpa Jalkanen, Anne Roivainen

**Affiliations:** 1 Turku PET Centre, University of Turku, Turku, Finland; 2 Department of Orthopaedics and Traumatology, Turku University Hospital and University of Turku, Turku, Finland; 3 Turku PET Centre, Turku University Hospital, Turku, Finland; 4 Department of Medical Physics, Turku University Hospital, Turku, Finland; 5 Department of Oncology and Radiotherapy, Turku University Hospital, Turku, Finland; 6 Department of Rheumatology and Clinical Immunology, Division of Medicine, Turku University Hospital, Turku, Finland; 7 Department of Radiology, Turku University Hospital, Turku, Finland; 8 MediCity Research Laboratory, University of Turku, Turku, Finland; and; 9 Heart Center, Turku University Hospital, Turku, Finland

**Keywords:** dosimetry, ^68^Ga, kinetics, PET, vascular adhesion protein 1, whole-body distribution

## Abstract

Sialic acid–binding immunoglubulinlike lectin 9 (Siglec-9) is a ligand of vascular adhesion protein 1. A ^68^Ga-labeled peptide of Siglec-9, ^68^Ga-DOTA-Siglec-9, holds promise as a novel PET tracer for imaging of inflammation. This first-in-humans study investigated the safety, tolerability, biodistribution, and radiation dosimetry of this radiopharmaceutical. **Methods:** Six healthy men underwent dynamic whole-body PET/CT. Serial venous blood samples were drawn from 1 to 240 min after intravenous injection of 162 ± 4 MBq of ^68^Ga-DOTA-Siglec-9. In addition to γ-counting, the plasma samples were analyzed by high-performance liquid chromatography to detect intact tracer and radioactive metabolites. Radiation doses were calculated using the OLINDA/EXM software, version 2.2. In addition, a patient with early rheumatoid arthritis was studied with both ^68^Ga-DOTA-Siglec-9 and ^18^F-FDG PET/CT to determine the ability of the new tracer to detect arthritis. **Results:** ^68^Ga-DOTA-Siglec-9 was well tolerated by all subjects. ^68^Ga-DOTA-Siglec-9 was rapidly cleared from the blood circulation, and several radioactive metabolites were detected. The organs with the highest absorbed doses were the urinary bladder wall (0.38 mSv/MBq) and kidneys (0.054 mSv/MBq). The mean effective dose was 0.022 mSv/MBq (range, 0.020–0.024 mSv/MBq). Most importantly, however, ^68^Ga-DOTA-Siglec-9 was comparable to ^18^F-FDG in detecting arthritis. **Conclusion:** Intravenous injection of ^68^Ga-DOTA-Siglec-9 was safe and biodistribution was favorable for testing of the tracer in larger group of patients with rheumatoid arthritis, as is planned for the next phase of clinical trials. The effective radiation dose of ^68^Ga-DOTA-Siglec-9 was within the same range as the effective radiation doses of other ^68^Ga-labeled tracers. Injection of 150 MBq of ^68^Ga-DOTA-Siglec-9 would expose a subject to 3.3 mSv. These findings support the possible repeated clinical use of ^68^Ga-DOTA-Siglec-9, such as in trials to elucidate the treatment efficacy of novel drug candidates.

## Visual Abstract

**Figure:**
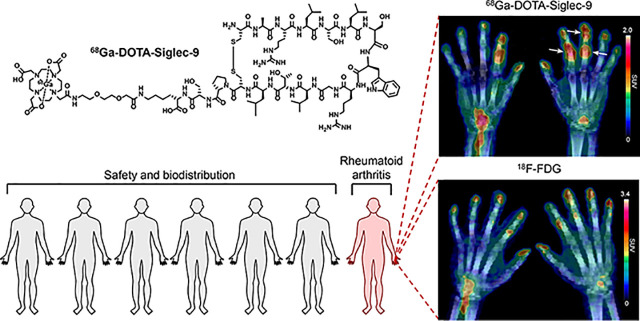

Early detection of inflammatory foci is critical for effective treatment of patients with a variety of diseases. Quantitative PET/CT imaging provides a valuable tool for diagnosing and monitoring the effects of therapeutic interventions. The glucose analog ^18^F-FDG is the gold standard radiopharmaceutical for inflammation using PET, but it is not specific. Also, ^68^Ga-citrate and ^89^Zr-transferrin can detect inflammation due to increased expression of transferrin receptor 1 on mononuclear cells, but the receptor is also expressed in cancer cells (1). Accordingly, radiopharmaceuticals more specific than these are needed to assess inflammation and its consequences for various pathologies.

Vascular adhesion protein 1 (VAP-1), also known as amine oxidase copper-containing 3, is an inflammation-inducible endothelial cell molecule that mediates leukocyte trafficking from blood to sites of inflammation. Although VAP-1 plays important roles in early phases of inflammation, its luminal expression on the endothelium remains constant if inflammation continues, making it a promising target for molecular imaging of inflammation. We previously showed that sialic acid-binding immunoglobulinlike lectin 9 (Siglec-9) is a VAP-1 ligand and that the ^68^Ga-labeled DOTA-conjugated peptide containing residues 283–297 of Siglec-9 (^68^Ga-DOTA-Siglec-9) can be used for PET imaging of inflammation in various experimental models (2–9).

We are currently engaged in the clinical evaluation phase of the ^68^Ga-DOTA-Siglec-9 radiopharmaceutical. The purpose of this study was to obtain information on the safety, tolerability, and whole-body biodistribution and kinetics of ^68^Ga-DOTA-Siglec-9 after a single intravenous injection in healthy volunteers and in a patient with rheumatoid arthritis (RA). The study sought to assess the effective radiation dose and radiation exposure in critical target organs to evaluate the safety of this ^68^Ga-labeled PET ligand. In addition, we evaluated the in vivo stability and pharmacokinetics of ^68^Ga-DOTA-Siglec-9—information that will be important for quantifying VAP-1 receptor density in future clinical trials of patients with inflammatory diseases. Finally, we tentatively compared the ability of ^68^Ga-DOTA-Siglec-9 PET/CT with that of ^18^F-FDG PET/CT in detecting RA.

## MATERIALS AND METHODS

### Chemicals and Reagents

Good-manufacturing-practice–grade precursor DOTA-Siglec-9 was obtained from ABX Advanced Biomedical Compounds GmbH. All other reagents were purchased from commercial suppliers and were either synthesis-grade or analytic-grade.

### Preparation of ^68^Ga-DOTA-Siglec-9

Radiosynthesis was performed as previously described using a fully automated synthesis device (Modular Lab PharmTracer; Eckert & Ziegler); the process complied with good-manufacturing-practice requirements (10). ^68^Ga was obtained from a ^68^Ge/^68^Ga generator (IGG-100, 1.85 GBq; Eckert and Ziegler) by eluting the generator with 6 mL of 0.1 M HCl and passing the eluate through a Strata-XC cation exchange cartridge. Bound ^68^GaCl_3_ was eluted with acidified acetone (0.8 mL, containing 3.25% water and 0.02 M HCl) into a reaction vial preloaded with a mixture of DOTA-Siglec-9 (30 μg, 12 nmol in 60 μL of water), sodium acetate buffer (2.0 mL, 0.2 M, pH 4.0), and absolute ethanol (0.2 mL). The reaction mixture was incubated at 65°C for 6 min and then diluted with saline (4 mL, 0.9 mg/mL). The crude product was purified by being loaded onto a C18 cartridge (SepPak Light C18; Waters) and by washing of the cartridge with saline (2 × 8 mL). ^68^Ga-DOTA-Siglec-9 was eluted with ethanol (1.3 mL, 70% [v/v]) through a nonpyrogenic 0.22-μm filter into the sterile final-product vial. The product was formulated in physiologic saline, and the final volume of the end product was 10 mL. The total synthesis time was 25 min.

The radiochemical purity of the product was evaluated by high-performance liquid chromatography (radio-HPLC) (LC-20A Prominence HPLC System [Shimadzu] and online radioactivity detector Flow-Count [Bioscan Inc.]) using an analytic Kinetex C18 column (2.6 μm, 100 Å, 75 × 4.6 mm; Phenomenex) at a flow rate of 1.0 mL/min and a gradient of 0.16% trifluoroacetic acid in water (A) and 0.16% trifluoroacetic acid in acetonitrile (B) (gradient from 18% B to 50% B over 12 min).

The in vitro stability of ^68^Ga-DOTA-Siglec-9 was tested in the formulation solution (saline) by incubation for 2 h at room temperature, followed by radio-HPLC analysis as described above.

### Healthy Subjects

Six healthy male volunteers (age, 37 ± 9 y; weight, 80 ± 4 kg; height, 181 ± 8 cm) were studied for the whole-body distribution kinetics of intravenously administered ^68^Ga-DOTA-Siglec-9 using PET/CT imaging. One catheter was inserted into an antecubital vein for injection of ^68^Ga-DOTA-Siglec-9, and another was inserted into the contralateral arm for blood sampling.

The study was approved by the joint Ethics Committee of the University of Turku and Turku University Hospital and by the Finnish Medicines Agency. Each subject gave informed consent before entering the study. The study was registered as a clinical trial (NCT03755245).

Absence of significant medical, neurologic, and psychiatric history, and of a history of alcohol or drug abuse, were assessed using questionnaires. In addition, a medical history review, routine blood tests, electrocardiography, and a physical exam were performed for each subject. During imaging, vital signs were monitored, including 12-lead electrocardiography, and blood and urine analyses were performed before and after PET/CT.

### RA Patient

Gadolinium-enhanced MRI, ^68^Ga-DOTA-Siglec-9 PET/CT, and ^18^F-FDG PET/CT were performed on a 49-y-old man with RA (duration of disease, 3.5 wk; rheumatoid factor–positive; anticitrullinated peptide antibodies, >340 U/mL; erythrocyte sedimentation rate, 5 mm/h; C-reactive protein, <1 mg/L). The patient fulfilled the 2010 American College of Rheumatology/European League Against Rheumatism RA classification criteria for his diagnosis. Disease activity was assessed clinically at screening, 4 wk before the PET studies, when disease activity score in 28 joints was 3.3 and the tender and swollen joint counts were 5.

### PET/CT

The biodistribution of ^68^Ga-DOTA-Siglec-9 was imaged using a Discovery 690 whole-body PET/CT scanner (GE Healthcare). Low-dose CT (120 kV, 14 mA) for attenuation correction was performed before PET.

In healthy subjects, ^68^Ga-DOTA-Siglec-9 (162 ± 4 MBq, 4.2 ± 0.9 mL, 13.6 ± 3.0 μg) formulated in saline was injected intravenously, and whole-body PET scanning started at 1, 10, 20, 40, 100, and 200 min after injection. The acquisition times per bed position were 30, 60, 120, 180, 300, and 360 s. Scanning at 100 min after injection included 14 bed positions, covering the head to the toes. All other scans used only 8 bed positions, covering the head to the mid thighs.

The patient with RA first underwent a ^18^F-FDG PET/CT whole-body scan. After the patient had fasted for 6 h, the PET scan started 46 min after a 198-MBq injection of ^18^F-FDG. An immobilization system was used for the hands and wrists to ensure the same positioning for the subsequent PET/CT. The patient was scanned from head to toes, with a 2-min acquisition time for each bed position. On the next day, the patient was intravenously injected with 175 MBq of ^68^Ga-DOTA-Siglec-9, and a 30-min dynamic PET/CT scan (time frames: 4 × 30 s, 3 × 60 s, 5 × 180 s, and 2 × 300 s) was performed on the hands, followed by a whole-body scan (3 min per bed position).

PET images were reconstructed using a 3-dimensional VUE Point algorithm with 2 iterations, 24 subsets, and a postprocessing filter of 3 mm in full width at half maximum. Scatter correction, random counts, and dead-time corrections were all incorporated into the reconstruction algorithm.

### Distribution Kinetics and Dose Estimates

Whole-body image data were quantified in accordance with the Radiation Dose Assessment Resource method for internal dose estimation (11). Time–activity values were determined for brain, bone (cortical and trabecular), heart contents (left ventricle), heart wall, kidneys, liver, lungs, muscle, pancreas, red bone marrow, salivary glands, spleen, and urinary bladder. Volumes of interest covered the whole organ or a representative volume of the organ. Urinary clearance and biologic half-life were estimated using measurements of urinary voids.

The resultant kinetic data were modeled using the sums of exponentials to determine the number of disintegrations (residence times) of the source organs. A urinary bladder voiding interval of 3.5 h was used in calculation of bladder residence time. Absorbed radiation doses were determined using these residence times and the OLINDA/EXM software, version 2.2 (12). An adult male (∼70 kg) reference model was used.

### Assessment of Arthritis

Volumes of interest were defined on the area of 3 inflamed finger joints (proximal interphalangeal joints 2 and 3 and distal interphalangeal joint 3) of the right hand. The time–activity curves of ^68^Ga-DOTA-Siglec-9 were determined, and the SUVs of ^68^Ga-DOTA-Siglec-9 and ^18^F-FDG were compared.

### Blood Analyses

Venous blood samples were collected into heparinized tubes at 2, 3, 5, 6, 7, 10, 15, 20, 30, 60, 90, 180, and 240 min the after injection of ^68^Ga-DOTA-Siglec-9.

The radioactivity of whole blood was measured with an automatic γ-counter (1480 Wizard 3″; EG&G Wallac). Plasma was separated by centrifugation (2,100*g* for 5 min at 4°C), and plasma radioactivity was measured. Then, 500 μL of plasma were mixed with 700 μL of acetonitrile to precipitate plasma proteins. The ratio of radioactivity in plasma versus blood and the percentage of radioactivity bound to plasma proteins were calculated as previously described (13).

To examine the stability of ^68^Ga-DOTA-Siglec-9 in vivo, 600 μL of plasma were mixed with 600 μL of 10% sulfosalicylic to precipitate plasma proteins. The protein-free plasma supernatants obtained after centrifugation were analyzed by radio-HPLC. Radio-HPLC was performed using a semipreparative Kinetex C18 column (5 μm, 300 Å, 150 × 10 mm; Phenomenex) at a flow rate of 5 mL/min and a gradient of 0.16% trifluoroacetic acid in water (A) and 0.16% trifluoroacetic acid in acetonitrile (B) as follows: 0−11 min, solvent B from 0% to 50%; 11−12 min, solvent B from 50% to 100%; 12−14 min, solvent B 100%; and 14−15 min, solvent B from 100% to 0%. The radio-HPLC system consisted of a LaChrom Instruments HPLC system (Hitachi) and a Radiomatic 150TR flow-through radioisotope detector (Packard). Urine samples were analyzed using the same HPLC procedure as for the plasma samples.

To estimate the plasma concentration of unchanged tracer, the fraction of ^68^Ga-metabolites in plasma was subtracted from the total radioactivity. The metabolite-corrected plasma concentration was used to calculate the pharmacokinetic parameters. Before any pharmacokinetic evaluation, all radioactivity values in blood and plasma were decay-corrected using the ^68^Ga physical half-life of 68 min. Pharmacokinetic parameters were obtained using monoexponential fitting of the tail and were adjusted for injected radioactivity dose.

### Soluble VAP-1

The activity of soluble VAP-1 in the plasma samples was measured using an assay described in detail previously (14). The levels of soluble VAP-1 in the heparin samples were measured with an in-house enzyme-linked immunosorbent assay as previously described (15).

### Statistical Analyses

Arithmetic mean values were calculated from the individual measurements and expressed at a precision of 1 SD (mean ± SD).

## RESULTS

### Preparation of ^68^Ga-DOTA-Siglec-9

The radiopharmaceutical ^68^Ga-DOTA-Siglec-9 was obtained at high yield (89.7% ± 2.0%, *n* = 6). Radioactivity concentration and molar activity at the end of synthesis were 56.7 ± 10.6 MBq/mL and 43.2 ± 8.0 GBq/μmol, respectively. Radiochemical purity was at least 95% in all batches, and the tracer remained radiochemically stable for 2 h (longer times were not tested) in sterile saline at room temperature. Quality control data from 3 representative batches of ^68^Ga-DOTA-Siglec-9 are shown in Supplemental Table 1 (supplemental materials are available at http://jnm.snmjournals.org).

### Safety and Tolerability

^68^Ga-DOTA-Siglec-9 was well tolerated by all subjects. We observed no adverse or clinically detectable pharmacologic effects in any subject. Moreover, we observed no significant changes in vital signs or in the results of laboratory studies or electrocardiograms. Healthy subject 6 was nauseous and got a headache at the end of PET/CT imaging, but it turned out that this subject had been fasting without water, contrary to the instructions, for a long period. The hematology, serology, and clinical chemistry of the study subjects are shown in Supplemental Tables 2 and 3.

### Whole-Body Distribution and Radiation Dose

**FIGURE 1.**
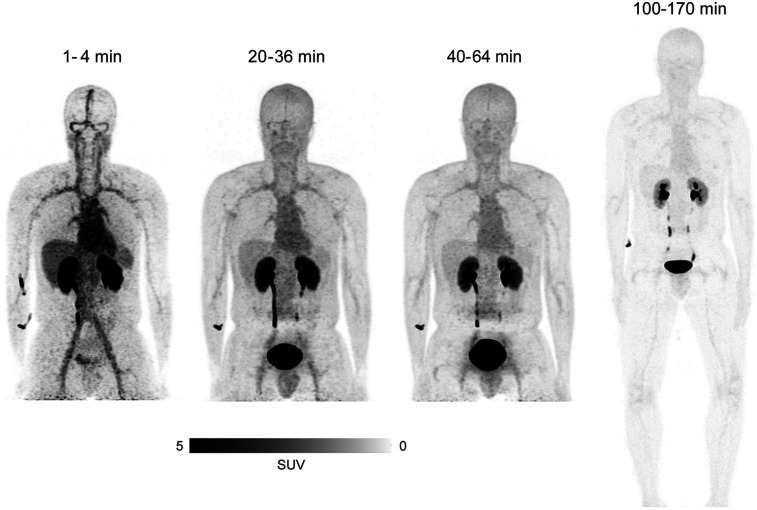
Whole-body coronal PET images of healthy volunteer (male; age, 29 y; weight, 74 kg; height, 183 cm) after intravenous injection of 167 MBq of ^68^Ga-DOTA-Siglec-9. Distribution of ^68^Ga-radioactivity 1–4 min after injection, based on imaging for 30 s per bed position, revealed high uptake, predominantly in heart, liver, kidneys, and ureter. At 20–36 min after injection and subsequent times, uptake was mainly in kidneys, ureter, and urinary bladder.

**FIGURE 2.**
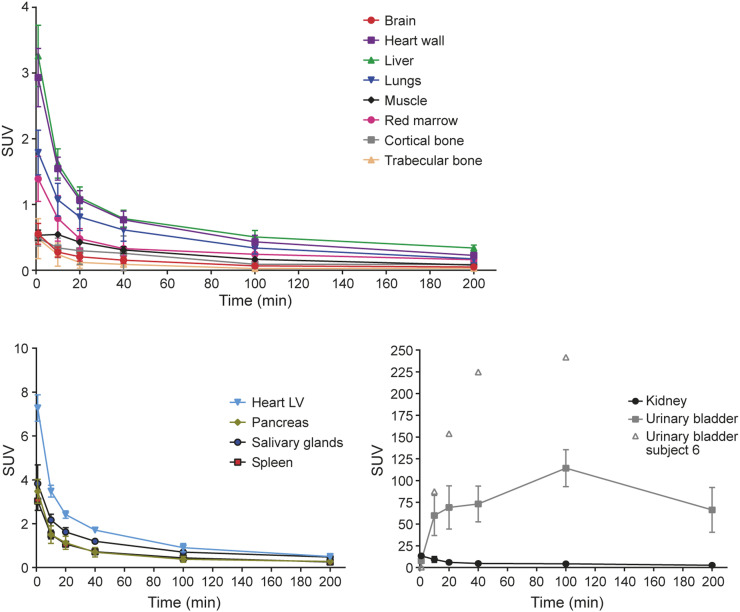
Decay-corrected time–activity curves of main organs. Result for urinary bladder in subject 6 was outlier so is presented separately. LV = left ventricle.

Whole-body PET/CT imaging of 5 healthy volunteers revealed that the ^68^Ga radioactivity after intravenous injection of ^68^Ga-DOTA-Siglec-9 was rapidly excreted through the kidneys to the urinary bladder (Fig. 1). The peak uptake of total injected radioactivity was highest in the urinary bladder contents, with an SUV of 114 (Fig. 2; Supplemental Fig. 1). Subject 6 differed from the other participants, exhibiting even higher urinary bladder radioactivity, peaking at an SUV of 242. The average biologic half-life of ^68^Ga-DOTA-Siglec-9 was 191 ± 33 min.

The normalized number of disintegrations (residence times) of the source organs and the remainder of the body are listed in Table 1. The largest mean residence times for ^68^Ga-DOTA-Siglec-9 were in the urinary bladder contents (0.33 h) and the remainder of the body (0.73 h).

**TABLE 1:** Normalized Number of Disintegrations (Hours) in Source Organs After Injection of ^68^Ga-DOTA-Siglec-9

Site	Mean	SD	Minimum	Maximum
Bone, cortical	0.0260	0.0045	0.0195	0.0312
Bone, trabecular	0.0039	0.0017	0.0025	0.0064
Brain	0.0047	0.0013	0.0033	0.0069
Heart contents	0.0033	0.0003	0.0030	0.0036
Kidneys	0.0359	0.0104	0.0231	0.0485
Liver	0.0377	0.0055	0.0266	0.0409
Lungs	0.0133	0.0033	0.0105	0.0176
Pancreas	0.0017	0.0004	0.0012	0.0021
Red marrow	0.0099	0.0019	0.0069	0.0127
Salivary gland	0.0005	0.0001	0.0005	0.0006
Spleen	0.0033	0.0004	0.0029	0.0040
Urinary bladder	0.3252	0.0426	0.2662	0.3831
Remainder of body	0.7257	0.0906	0.5861	0.8501

Estimates of absorbed doses, reported in Table 2, were based on a male adult weighing 70 kg. The organs with the highest absorbed doses were the urinary bladder wall (383.2 μSv/MBq) and the kidneys (54.4 μSv/MBq). The mean effective dose (International Commission on Radiological Protection publication 103) (16) was 0.022 mSv/MBq. Thus, the effective dose from 150 MBq of injected radioactivity was 3.3 mSv.

**TABLE 2:** Dose-Equivalent Estimates (μSv/MBq) and Effective Dose (mSv/MBq) After Injection of ^68^Ga-DOTA-Siglec-9

Site	Mean	SD	Minimum	Maximum
Adrenals	12.2	1.4	10.7	14.2
Brain	2.4	0.5	1.9	3.3
Colon, left	8.3	0.8	7.2	9.3
Colon, right	8.4	0.8	7.3	9.4
Esophagus	6.9	0.8	5.9	8.0
Eyes	5.9	0.7	4.8	6.9
Gallbladder wall	8.7	0.9	7.7	9.8
Heart wall	11.5	1.3	10.0	13.3
Kidneys	54.4	14.9	36.0	72.5
Liver	12.7	1.6	9.4	13.7
Lungs	6.8	1.2	5.8	8.5
Osteogenic cells	9.9	1.2	8.4	11.7
Pancreas	8.5	1.4	6.8	10.0
Prostate	17.4	0.5	16.7	17.9
Rectum	14.1	0.1	14.0	14.3
Red marrow	8.6	0.8	7.7	10.0
Salivary glands	4.5	0.3	4.0	5.0
Small intestine	9.5	0.6	8.5	10.3
Spleen	12.8	1.2	11.5	14.6
Stomach wall	7.5	0.8	6.4	8.7
Testes	8.8	0.5	8.0	9.5
Thymus	6.8	0.8	5.7	8.0
Thyroid	6.7	0.8	5.5	7.8
Urinary bladder wall	383.2	48.5	316.0	449.0
Whole body	10.1	0.5	9.4	10.7
Effective dose*	0.022	0.002	0.020	0.024

* International Commission on Radiological Protection publication 103 (16).

### Assessment of Arthritis

**FIGURE 3.**
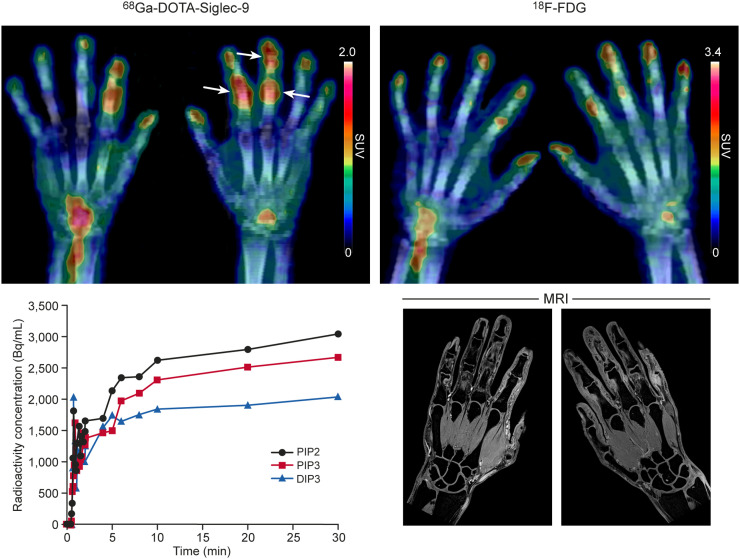
^68^Ga-DOTA-Siglec-9 and ^18^F-FDG PET/CT and MR images of hands of 49-y-old patient with early RA. Arrows denote proximal interphalangeal joints PIP2 and PIP3 and distal interphalangeal joint DIP3, whose time–activity curves are at bottom left. Respective SUVs were 1.05, 0.92, and 0.70 with ^68^Ga-DOTA-Siglec-9 and 1.46, 1.22. and 1.08 with ^18^F-FDG.

High regional uptake of both ^68^Ga-DOTA-Siglec-9 and ^18^F-FDG was observed at the site of inflamed joints as compared with the unaffected joints. The time course at the site of arthritis revealed very fast uptake of ^68^Ga-DOTA-Siglec-9, which plateaued after 10 min (Fig. 3).

### Metabolism and Pharmacokinetics

**FIGURE 4.**
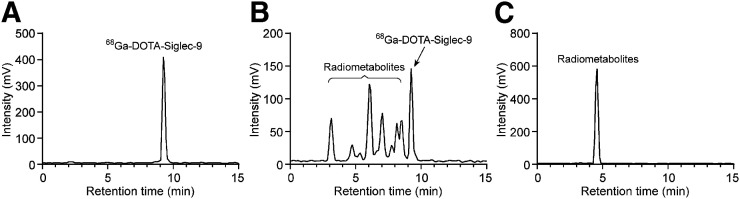
Representative radio-HPLC chromatograms of intact ^68^Ga-DOTA-Siglec-9 (A), human plasma 5 min after injection (B), and urine at 40 min after injection (C). In addition to parent tracer, several other peaks were detected in plasma, whereas in urine, radioactivity was entirely from radiometabolite.

After administration of ^68^Ga-DOTA-Siglec-9, the concentration of radioactivity in venous plasma declined rapidly, and several other peaks in addition to the intact tracer were detected by radio-HPLC. Typical radio-HPLC chromatograms are shown in Figure 4 and Supplemental Figure 2. The retention time of ^68^Ga-DOTA-Siglec-9 was 9.3 ± 0.1 min. According to the radio-HPLC analysis, the mean ± SD of intact ^68^Ga-DOTA-Siglec-9 were 79.2% ± 5.3% and 4.3% ± 1.6% at 1 and 10 min after injection, respectively. Two radiometabolite peaks, with retention times of 3.2 ± 0.1 min and 4.6 ± 0.1 min, were still detectable in plasma at 90 min after injection. Analysis of urine revealed only 1 radiometabolite peak (retention time, 4.6 ± 0.1 min) in all patients.

**FIGURE 5.**
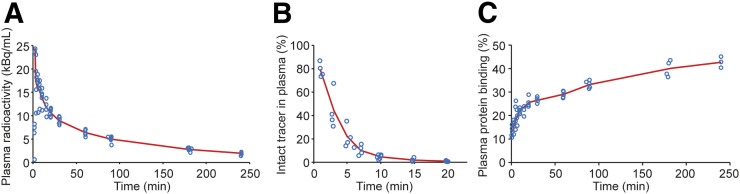
(A and B) Total radioactivity (A) and proportion of parent tracer (B) decreased rapidly in blood circulation. (C) On average, 26% of radioactivity was bound to plasma proteins.

The pharmacokinetic parameters determined for total radioactivity and the intact tracer are summarized in Supplemental Table 4. Time–activity curves revealed rapid clearance from the blood circulation (Fig. 5A), and the PET image–derived curves from heart left ventricle closely correlated with radioactivity concentration measured from venous blood (Supplemental Fig. 3). The mean plasma half-life of total radioactivity was 106.9 min, and total clearance was 0.18 mL/min. For the intact tracer, total clearance was estimated to be 3.3 mL/min (Fig. 5B).

The plasma-to-blood ratio of radioactivity was 1.7 ± 0.04 throughout the 240-min duration of the PET imaging, indicating that most of the radioactivity was in the plasma. Mean plasma protein binding of radioactivity was 25.9% ± 8.8%; the value slowly increased during PET imaging, ranging from 13.3% to 42.7% (Fig. 5C).

### Soluble VAP-1

Because VAP-1 is also present in a soluble form in plasma, we also analyzed the concentration of soluble VAP-1 protein and its enzymatic activity in plasma. On the day of PET imaging, soluble VAP-1 levels in plasma were 874.0 ± 139.9 ng/mL, and enzymatic activity levels were 10.9 ± 2.2 nmol/L/h, as determined from 4 healthy subjects.

## DISCUSSION

^68^Ga-DOTA-Siglec-9 is a promising novel PET tracer for the imaging of inflammation. Here, we report the whole-body distribution and radiation exposure of ^68^Ga-DOTA-Siglec-9. We also evaluated its metabolic fate and pharmacokinetics in healthy humans. Most importantly, however, we show for the first time that ^68^Ga-DOTA-Siglec-9 PET/CT clearly delineates arthritis in a pilot patient with early RA.

Recently, we set up a well-validated radiosynthesis protocol for the production of good-manufacturing-practice–grade ^68^Ga-DOTA-Siglec-9 (10). This protocol is highly reproducible, and to date the successful production rate in our lab has been 100%. This has paved the way for evaluation of ^68^Ga-DOTA-Siglec-9 in the clinical setting. Accordingly, following the same radiosynthesis protocol, in this study we produced 6 batches of ^68^Ga-DOTA-Siglec-9 with high radiochemical yield, high radiochemical purity, and high molar activity. Before clinical trials, we performed safety studies of DOTA-Siglec-9 in rats, complying with the requirements of good manufacturing practices and good laboratory practices and using a 1,000-fold excess of the planned clinical dose (40 μg per subject) (17). In routine radiosynthesis, we managed to use even less precursor (30 μg) in each batch because of the efficiency of the synthesis protocol; the actual clinical dose (13.6 ± 3.0 μg per subject) is far below the estimated clinical dose used in the safety evaluations. Therefore, the potential clinical risks (if any) related to the test item itself were further reduced for this first-in-humans trial.

Intravenously administered ^68^Ga-DOTA-Siglec-9 exhibited fast renal clearance; this is presumably connected to the hydrophilic properties and small size of the peptide. The highest radioactivity uptake was in the urinary bladder, in line with the previous preclinical studies with Siglec-9 peptide (7), which could be reduced with frequent bladder voids. The radioactive metabolites of ^68^Ga-DOTA-Siglec-9 may contribute to uptake in the kidneys and urinary bladder, although the extent of such uptake remains unknown.

The results of image quantification and the estimates of radiation dose were consistent across the subject population, except for one subject who had significantly higher urinary bladder uptake than the others. Notably, this subject had been fasting without water, contrary to the instructions, for a long period. Although ^68^Ga has been used extensively for the labeling of synthetic peptides, data for human dose estimates are available for only a few, such as for peptide analogs that bind to somatostatin receptors, bombesin, or α_v_β_3_ integrin. The effective dose of ^68^Ga-DOTA-Siglec-9 reported here (0.022 mSv/MBq according to International Commission on Radiological Protection publication 103) is within the range for ^68^Ga-DOTATOC (0.023 mSv/MBq), ^68^Ga-DOTANOC (0.025 mSv/MBq), and ^68^Ga-NOTA-RGD (0.022 mSv/MBq) (18–20).

Although only one patient has been studied so far, the most intriguing finding in this study is the ability of ^68^Ga-DOTA-Siglec-9 PET/CT to detect early RA. The observed SUVs of ^68^Ga-DOTA-Siglec-9 (0.70−1.05) were slightly lower than those of ^18^F-FDG (1.08−1.46), but it remains to be seen how optimization of the imaging protocol would affect ^68^Ga-DOTA-Siglec-9 results.

^68^Ga-DOTA-Siglec-9 exhibited rapid blood clearance, which is usually due to fast renal excretion. In addition to intact ^68^Ga-DOTA-Siglec-9, several additional peaks were observed in plasma radio-HPLC chromatograms, but we did not further identify the radiometabolites of plasma or investigate their extent at the inflammatory sites. Radiometabolism and plasma protein binding are consistent with values reported previously in pigs (6,21).

Blood radioactivity concentration did not correlate with soluble VAP-1 level. Soluble VAP-1 levels in the plasma of healthy subjects varied from 729 to 1,082 ng/mL, with a median of 874 ng/mL. These results are within the range previously reported in a Finnish prospective cohort study (15). The enzymatic activity levels of soluble VAP-1 reported here are slightly lower than those of previous studies. The lower values can be explained by our use of heparin-plasma samples, which have been shown to interfere with soluble VAP-1 enzymatic activity in comparison to serum samples (14).

Radiation burden and biodistribution were comparable to earlier results extrapolated from rats (7). The difference between the effective dose derived from rat data (0.024 mSv/MBq) and the human dosimetry (0.022 mSv/MBq) is small and, in practice, within the limits of measurement accuracy.

## CONCLUSION

Intravenously injected ^68^Ga-DOTA-Siglec-9 was safe and rapidly cleared from the blood by renal excretion. The radiation burden from ^68^Ga-DOTA-Siglec-9 was relatively low and comparable to other ^68^Ga-peptides. Frequent urination is recommended to reduce the radiation dose to the bladder. From a radiation safety perspective, ^68^Ga-DOTA-Siglec-9 imaging is feasible for applied clinical studies and could be performed in the same subject multiple times per year. Such repeatability would be useful in, for example, trials aiming to clarify the treatment efficacy of novel drug candidates. On the basis of these results, we conclude that ^68^Ga-DOTA-Siglec-9, which targets VAP-1, represents a promising new PET radiopharmaceutical for imaging inflammation in such diseases as RA.

## DISCLOSURE

Sirpa Jalkanen owns stocks in Faron Pharmaceuticals Ltd. The study was conducted within the Finnish Centre of Excellence in Cardiovascular and Metabolic Diseases, supported by the Academy of Finland, University of Turku, Turku University Hospital, and Åbo Akademi University. The study was financially supported by grants from the Academy of Finland, the State Research Funding of Turku University Hospital, Business Finland, the Sigrid Jusélius Foundation, the Jane and Aatos Erkko Foundation, the Finnish Foundation for Cardiovascular Research, the Finnish Cultural Foundation, and the Drug Research Doctoral Program of the University of Turku Graduate School. No other potential conflict of interest relevant to this article was reported.

**KEY POINTS**

**QUESTION:** Is the new radiopharmaceutical ^68^Ga-DOTA-Siglec-9 safe, and how is it distributed in healthy humans and patients with RA?
**PERTINENT FINDINGS:** Six healthy men and a patient with RA were studied by whole-body PET/CT imaging with safety monitoring and analyses of blood and urine. ^68^Ga-DOTA-Siglec-9 was safe and cleared rapidly from the blood circulation via renal excretion into the urine. ^68^Ga-DOTA-Siglec-9 clearly detected arthritic joints. The highest radiation exposure was to the urinary bladder wall and kidneys. The effective dose, 0.022 mSv/MBq, was comparable to those of other ^68^Ga tracers.
**IMPLICATIONS FOR PATIENT CARE:** The characteristics of ^68^Ga-DOTA-Siglec-9 are favorable for planned patient studies.
