# Supplementary material for: First-in-Humans Study of 68Ga-DOTA-Siglec-9, a PET Ligand Targeting Vascular Adhesion Protein 1
Source: J Nucl Med. 2021 Apr;62(4):577–83. doi: 10.2967/jnumed.120.250696 (PMC8049366; doi:10.2967/jnumed.120.250696)
Supplement: Supplementary file 1 [file jnm250696SupplementaryData.pdf]

SUPPLEMENTAL DATA

First-in-Human Study of ^{68}Ga-DOTA-Siglec-9, PET Ligand Targeting Vascular Adhesion Protein 1

Riikka Viitanen[1], Olli Moisio[1], Petteri Lankinen[2,3], Xiang-Guo Li[1], Mikko Koivumäki[3], Sami Suilamo[4,5], Tuula Tolvanen[3,4], Kirsi Taimen[6], Markku Mali[6], Ia Kohonen[7], Ilpo Koskivirta[6], Vesa Oikonen[1], Helena Virtanen[1], Kristiina Santalahti[8], Anu Autio[1,8], Antti Saraste[1,3,9], Laura Pirilä[6], Pirjo Nuutila[1,3], Juhani Knuuti[1,3], Sirpa Jalkanen[8], and Anne Roivainen[1,3]

[1]Turku PET Centre, University of Turku, Turku, Finland; [2]Department of Orthopaedics and Traumatology, Turku University Hospital and University of Turku, Turku, Finland; [3]Turku PET Centre, Turku University Hospital, Turku, Finland; [4]Department of Medical Physics, Turku University Hospital, Turku, Finland; [5]Department of Oncology and Radiotherapy, Turku University Hospital, Turku, Finland; [6]Department of Rheumatology and Clinical Immunology, Division of Medicine, Turku University Hospital, Turku, Finland; [7]Department of Radiology, Turku University Hospital, Turku, Finland; [8]MediCity Research Laboratory, University of Turku, Turku, Finland; [9]Heart Center, Turku University Hospital, Turku, Finland

Correspondence: Prof. Anne Roivainen, PhD, Turku PET Centre, Kiinamyllynkatu 4-8, FI-20521 Turku, Finland. Tel +35823132862, Fax +35822318191, E-mail anne.roivainen@utu.fi

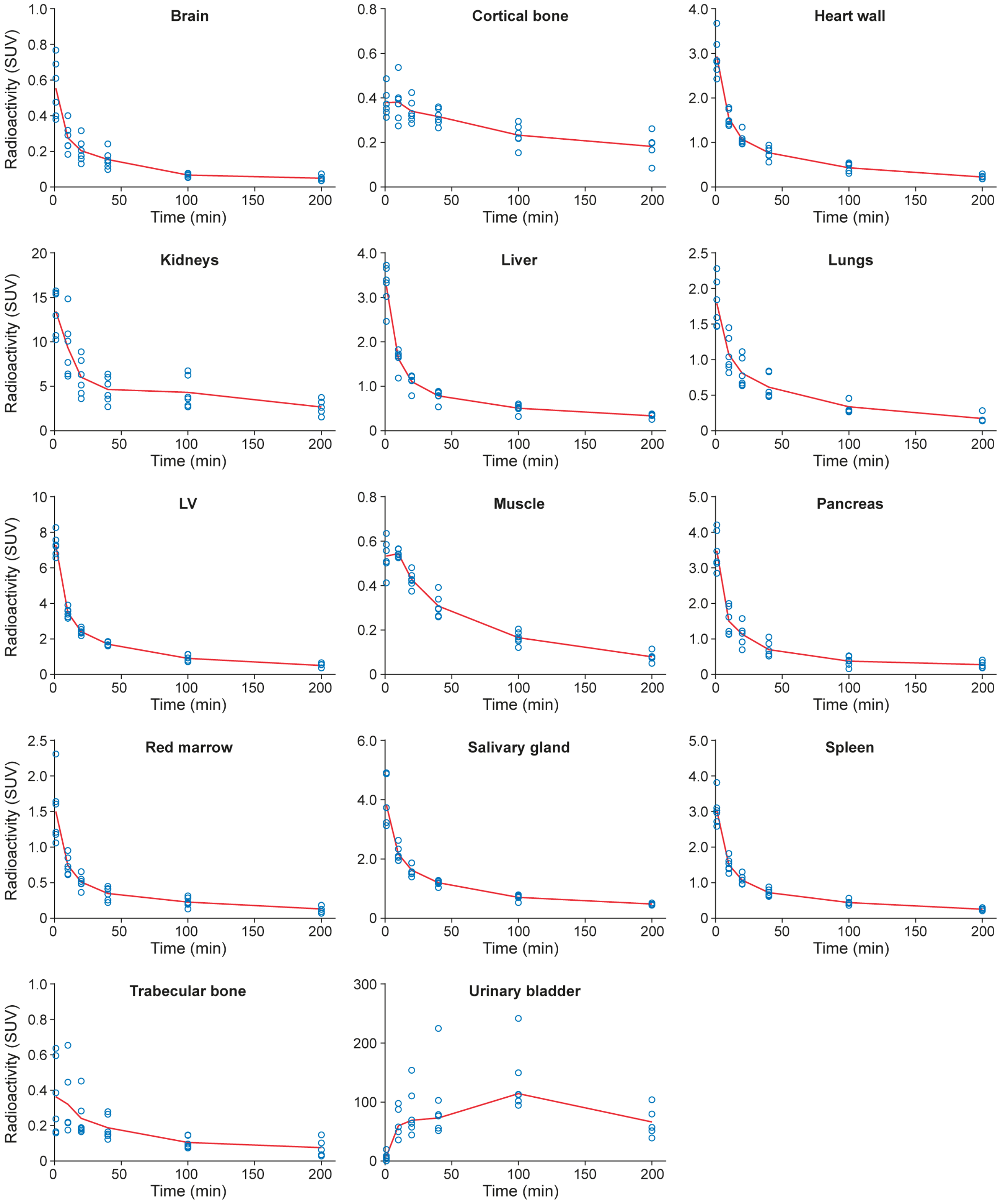

SUPPLEMENTAL FIGURE 1. Decay-corrected time-activity curves of main organs of six healthy subjects. The open circles represent each individual and red line is the average. SUV = standardized uptake value; LV = heart left ventricle.

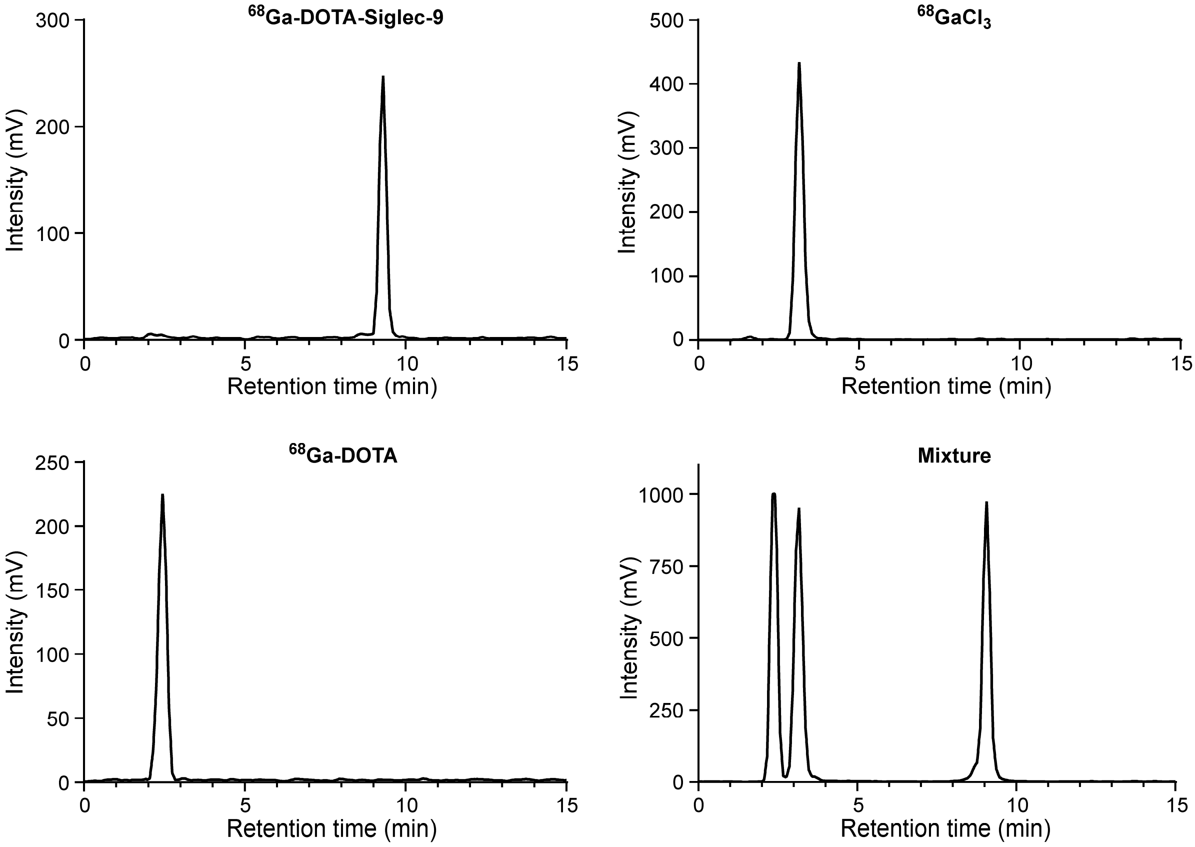

SUPPLEMENTAL FIGURE 2. Representative radio-HPLC chromatograms of authentic standards. Mixture = *in vitro* mixed ^{68}Ga-DOTA-Siglec-9, $^{68}GaCl_3$, and ^{68}Ga-DOTA.

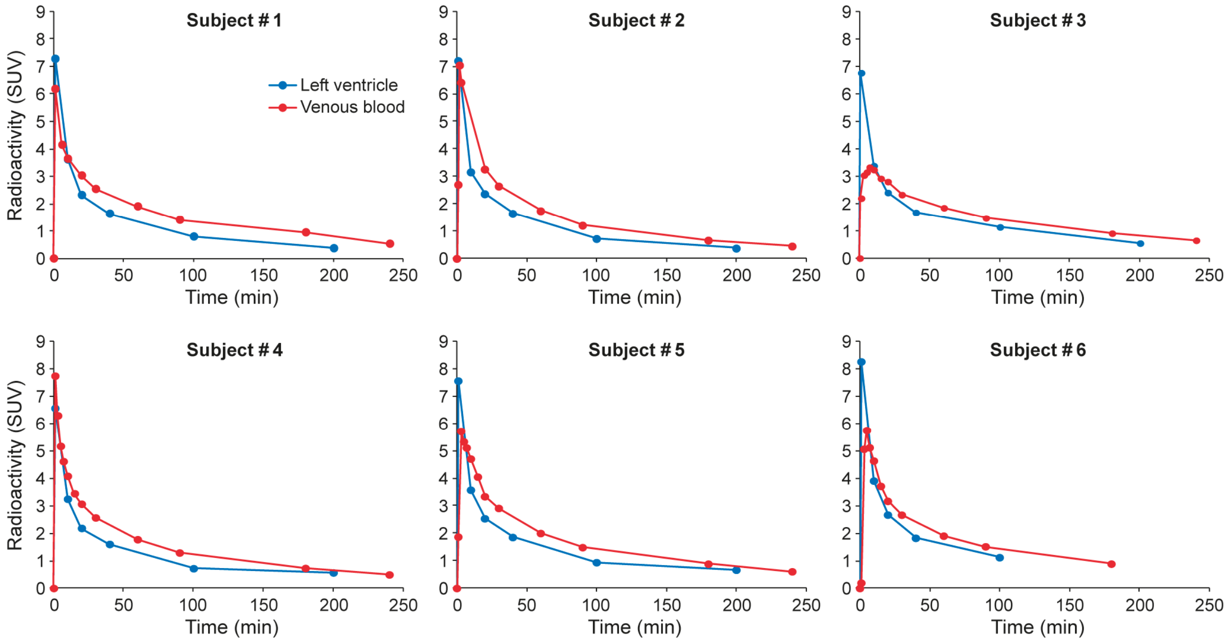

SUPPLEMENTAL FIGURE 3. PET image-derived time-activity curve from heart left ventricle closely correlates with radioactivity concentration of venous blood measured with a gamma counter. SUV = standardized uptake value.

SUPPLEMENTAL TABLE 1

Quality Control Data from Three Representative Batches of ^{68}Ga-DOTA-Siglec-9

Product specifications	Limits for product release	Results
Appearance	Clear and colorless solution, free of particles	Pass
pH	4.0 – 8.0	5.0 ± 0.0
Radionuclidic identity	68 min ± 3 min	67.7 ± 0.6 min
Identity	Retention time of ^{68}Ga-DOTA-Siglec-9 complies with the retention time of reference standard (± 1.0 min)	Pass*
Radiochemical purity	≥ 91% by HPLC	96.0 ± 1.0%
	≥ 91% by iTLC	97.7 ± 0.6%
Sum of unknown chemical impurities	≤ 4.1 µg/mL based on DOTA-Siglec-9	Pass
Content of DOTA-Siglec-9	≤ 4.1 µg/mL based on DOTA-Siglec-9	Pass
Radionuclidic purity	≥ 99.9% ^{68}Ga	Pass
	≤ 0.001% ^{68}Ge	Pass
Content of ethanol	≤ 10%	7.1 ± 0.7%
Residual solvents (acetone)	≤ 0.5%	0.0 ± 0.0%
Sterile filter integrity	≥ 3.45 bar	3.6 ± 0.1 bar
Sterility	Sterile (no viable organisms detected)	Pass
Bacterial endotoxins	≤ 17.5 endotoxin units/mL	Pass†

* Retention time of reference standard was 7.6 ± 0.1 min, and retention time of ^{68}Ga-DOTA-Siglec-9 was 7.7 ± 0.1 min.

† In each of the three batches of product, the level of bacterial endotoxins was < 0.1 units/mL.

SUPPLEMENTAL TABLE 2

Hematology, Serology, and Clinical Chemistry of Study Subjects

Parameter	Screening	Before PET/CT	After PET/CT	Reference value
White blood cell count (10^9/L)	5.8 ± 1.2	6.1 ± 1.1	7.7 ± 0.8	3.4 – 8.2
Red blood cell count (10^{12}/L)	5.1 ± 0.2	5.0 ± 0.2	4.8 ± 0.1	4.3 – 5.7
Hemoglobin (g/L)	154 ± 6.9	147 ± 2.9	145 ± 2.7	134 – 167
Hematocrit	0.45 ± 0.02	0.43 ±0.01	0.42 ± 0.01	0.39 – 0.50
Mean corpuscular volume (fL)	87 ± 2.3	87 ± 1.3	87 ± 0.7	82 – 98
Mean corpuscular hemoglobin (pg)	30 ± 0.8	30 ± 0.5	30 ± 0.3	27 – 33
Platelet count (10^9/L)	212 ± 41	202 ± 22	196 ± 15	150 – 360
Neutrophil count (10^9/L)	3.0 ± 1.1	3.5 ± 1.0	5.1 ± 0.9	1.5 – 6.7
Lymphocyte count (10^9/L)	2.0 ± 0.3	1.8 ± 0.1	1.9 ± 0.1	1.3 – 3.6
Monocyte count (10^9/L)	0.5 ± 0.1	0.6 ± 0.1	0.5 ± 0.1	0.2 – 0.8
Eosinophil count (10^9/L)	0.23 ± 0.17	0.18 ± 0.05	0.14 ± 0.05	0.03 – 0.44
Basophil count (10^9/L)	0.05 ± 0.03	0.05 ± 0.01	0.04 ± 0.06	0.0 – 0.1
Absolute neutrophils (%)	51 ± 9.6	54 ± 5.3	64 ± 4.6	41 – 81
Absolute lymphocytes (%)	35 ± 9.4	32 ± 5.0	26 ± 3.1	20 – 45
Absolute monocytes (%)	9.3 ± 1.2	9.8 ± 0.9	7.5 ± 1.0	1 – 11
Absolute eosinophils (%)	3.8 ± 2.2	3.6 ± 1.2	2.2 ± 0.8	1 – 5
Absolute basophils (%)	0.8 ± 0.4	1.0 ± 0.3	0.5 ± 0.2	0 – 1
Erythrocyte sedimentation rate (mm/h)	3.7 ± 2.9	3.2 ± 0.7	2.0 ± 0.0	< 15
C-reactive protein (mg/L)	1.2 ± 0.4	1.0 ± 0.0	1.0 ± 0.0	< 10
Potassium (mmol/L)	4.1 ± 0.1	3.9 ± 0.1	3.9 ± 0.1	3.3 – 4.8
Sodium (mmol/L)	142 ± 2.1	142 ± 0.5	142 ± 0.7	137 – 144
Creatinine (μmol/L)	88 ± 13	82 ± 5.5	80 ± 5.2	60 – 100
Alkaline phosphatase (U/L)	70 ± 12	65 ± 5.2	59 ± 5.0	35 – 105
Alanine aminotransferase (U/L)	44.7 ± 21.0	36.2 ± 7.6	35.7 ± 6.6	< 50
Rheumatoid factor (IU/mL)	< 10	ND	ND	< 14
Citrullinated peptide antibody (U/mL)	< 7	ND	ND	< 7

ND = not determined

SUPPLEMENTAL TABLE 3

Hematology, Serology, and Clinical Chemistry of a Patient with Rheumatoid Arthritis

Parameter	Before PET/CT	After PET/CT	Reference value
White blood cell count (10^9/L)	4.6	6.3	3.4 – 8.2
Red blood cell count (10^{12}/L)	4.50	4.23 *	4.3 – 5.7
Hemoglobin (g/L)	147	138	134 – 167
Hematocrit	0.41	0.39	0.39 – 0.50
Mean corpuscular volume (fL)	91	92	82 – 98
Mean corpuscular hemoglobin (pg)	33	33	27 – 33
Platelet count (10^9/L)	118 *	122 *	150 – 360
Neutrophil count (10^9/L)	3.02	4.67	1.5 – 6.7
Lymphocyte count (10^9/L)	0.96 *	1.03 *	1.3 – 3.6
Monocyte count (10^9/L)	0.52	0.50	0.2 – 0.8
Eosinophil count (10^9/L)	0.05	0.05	0.03 – 0.44
Basophil count (10^9/L)	0.03	0.03	0.0 – 0.1
Absolute neutrophils (%)	66	74	41 – 81
Absolute lymphocytes (%)	21	16 *	20 – 45
Absolute monocytes (%)	11	8	1 – 11
Absolute eosinophils (%)	1	1	1 – 5
Absolute basophils (%)	1	1	0 – 1
Erythrocyte sedimentation rate (mm/h)	5	7	< 15
C-reactive protein (mg/L)	< 1	< 1	< 10
Potassium (mmol/L)	3.9	4.0	3.3 – 4.8
Sodium (mmol/L)	139	140	137 – 144
Creatinine (µmol/L)	77	72	60 – 100
Alkaline phosphatase (U/L)	37	33 *	35 – 105
Alanine aminotransferase (U/L)	17	16	< 50
Rheumatoid factor (IU/mL)	19 *	ND	< 14
Anti-citrullinated peptide antibodies (U/mL)	> 340 *	ND	< 7

ND = not determined, * Not within the reference value

SUPPLEMENTAL TABLE 4

Plasma Pharmacokinetic Parameters After Intravenous Administration of ^{68}Ga-DOTA-Siglec-9

	Total radioactivity				Intact tracer	
Parameter	k_{el} (1/min)	$t_{1/2}$ (min)	$AUC_{(0\text{-}tlast)}$ (kBq*min/mL)	Cl_T (mL/min)	$AUC_{(0\text{-}tlast)}$ (kBq*min/mL) †	Cl_T (mL/min) †
Arithmetic mean	0.0065	106.9	915.9	0.1783	53.6	3.3221
Arithmetic SD	0.0004	7.1	86.3	0.0144	17.1	1.1450
Coefficient of variation (%)*	6.6	6.6	9.4	8.1	31.8	34.5

Injected dose of ^{68}Ga-DOTA-Siglec-9 was 162 ± 4 MBq (total mass, 13.6 ± 3.0 µg). Six experiments were performed.

k_{el} = elimination rate constant; $t_{1/2}$ = plasma half-life; AUC = area under curve; Cl_T = total clearance.

* SD/mean×100.

† Values have to be regarded as estimates because sampling time was not sufficient to adequately describe the terminal elimination phase.
